# Improving the performance of opportunistic routing using min-max range and optimum energy level for relay node selection in wireless sensor networks

**DOI:** 10.7717/peerj-cs.326

**Published:** 2020-12-07

**Authors:** Por Lip Yee, Shahid Mehmood, Ahmad Almogren, Ihsan Ali, Mohammad Hossein Anisi

**Affiliations:** 1 Department of Computer System and Technology, Faculty of Computer Science and Information Technology, University of Malaya, Kuala Lumpur, Malaysia; 2 Department of Computer Science, College of Computer and Information Sciences, King Saud University, Riyadh, Saudi Arabia; 3 School of Computer Science and Electronic Engineering, University of Essex, Colchester, United Kingdom

**Keywords:** Opportunistic routing, Optimum energy, Threshold energy level, Relay node, Wireless sensor networks (WSNs)

## Abstract

Opportunistic routing is an emerging routing technology that was proposed to overcome the drawback of unreliable transmission, especially in Wireless Sensor Networks (WSNs). Over the years, many forwarder methods were proposed to improve the performance in opportunistic routing. However, based on existing works, the findings have shown that there is still room for improvement in this domain, especially in the aspects of latency, network lifetime, and packet delivery ratio. In this work, a new relay node selection method was proposed. The proposed method used the minimum or maximum range and optimum energy level to select the best relay node to forward packets to improve the performance in opportunistic routing. OMNeT++ and MiXiM framework were used to simulate and evaluate the proposed method. The simulation settings were adopted based on the benchmark scheme. The evaluation results showed that our proposed method outperforms in the aspect of latency, network lifetime, and packet delivery ratio as compared to the benchmark scheme.

## Introduction

Opportunistic Routing (OR) is a routing scheme that takes advantage of the broadcasting nature of the wireless medium to improve the link reliability, efficiency, and the network throughput in multi-hop routing (Chakchouk, 2015; Eu, Tan & Seah, 2010; Jadhav & Satao, 2016).

According to Biswas & Morris (2005), Boukerche & Darehshoorzadeh (2014), Chachulski et al. (2007), Choudhury & Vaidya (2004) and Larsson (2001), OR improves network performance in the context of multi-hop and mesh networks, such as relay node selection in opportunistic networks. A multi-hop network is a network of relay nodes that are connected through the communication links. Due to the limited transmission range, the relay nodes in the network may not be able to communicate directly with the destination node. Hence, they need other relay nodes that can forward packets to the destination node (Zhao, Mosler & Braun, 2012).

In OR, the forwarder method selects the forwarder node that is nearer to the destination node to forward the packets (Jadhav & Satao, 2016). For the source node to forward the packets to the destination node, the OR forwarder method selects a next-hop which is determined by using a routing metric such as energy, geographical distance, hop count, expected transmission count (ETX), and expected transmission time (ETT). These routing metrics could be used to forward the packets (Menon, 2019). The source node constructs a list of forwarder nodes to transmit the packets to the destination node. This list is developed based on priority, and each relay node is selected based on the metrics per the OR forwarder method requirements (Biswas & Morris, 2005).

There are several advantages of using OR. Compared to legacy routing, OR avoids duplicate packet transmission, and it also reduces the amount of packet retransmission significantly due to link failures. OR can exploit the reception of a similar packet at multiple available relay nodes in order to improve the network performance especially in multi-hop and mesh wireless networks (Biswas & Morris, 2005; Chachulski et al., 2007; Choudhury & Vaidya, 2004; Larsson, 2001).

In multi-hop wireless networks, packets are forwarded via at least one intermediate relay node from the source node to the destination node (Coutinho & Boukerche, 2017). A mesh wireless network is referred as a network topology in which the infrastructure of the nodes is connected directly, dynamically, and non-hierarchically to the other nodes (Darehshoorzadeh, Robson & Boukerche, 2015; Li et al., 2019). Therefore, proposing an effective forwarder method to forward packets from one relay node to another in such networks is important because it will affect the performance of the networks (Jain, Dongariya & Verma, 2017).

Throughout the years, many forwarder methods have been proposed to improve the performance of OR. In general, most of the forwarder methods use routing metrics such as energy, geographical distance, hop count, ETX, and ETT to forward packets (Menon, 2019). However, these methods have several drawbacks, especially in the aspects of latency, first dead node, network lifetime, and receiving packets ratio. Several relay node selection methods as a means to improve the performance of routing in the opportunistic network. This study expands on the above recommendation by conducting a study that aims to improve the performance of routing in the opportunistic network.

In this research work, a relay node selection method that uses maximum and minimum range (min-max range) and optimum energy level to select the best forwarder node to improve the performance in OR is proposed. The simulation results showed that the proposed method could reduce the lowest latency, produced the highest time for the first dead node, improved the network lifetime, and produced a higher receiving packet ratio compared to the related works (AOR, ENO_OR, ENS_OR, EXOR, GeRaF, and EEOR).

## Related Works

In 2003, Zorzi & Rao (2003) proposed a forwarder method named Geographic Random Forwarding (GeRaF). This method is based on the geographical location of the nodes involved. Initially, the active relay nodes, which are located nearer to the destination node, will send a “clear to send” message to the source node. The source node then will discover the relay nodes that can participate in the packet forwarding process around its coverage area. During the discovery, the source node will receive an acknowledgement from each of the relay nodes that can participate in packet forwarding. The source node will randomly select one of the relay nodes as the forwarder node to forward the packet. The forwarder node will use the same mechanism to randomly select another forwarder node until the packet reaches the destination node. According to the authors, this method can reduce latency because it randomly selects a relay node that can forward a packet without further delay. However, the scheduling algorithm used in this method might produce a low network lifetime because it does not consider the energy level of the relay node when determining the forwarder node. Energy is an important aspect because if a relay node has a low energy level, it might die quickly, or it might drop the received packet due to insufficient energy.

Biswas & Morris (2005) proposed a forwarder method named Opportunistic Multi-hop Routing (ExOR) in 2005. According to the author, ExOR is one of the initial primary protocols, which practically implemented opportunistic routing in WSNs. In this method, packets deliver to the same destination are grouped in a batch by the source node. Each batch has a unique ID. In order to deliver the packets, the source node needs to determine a forwarder node based on distance and the ETX. Higher priority is given to the node, which has a shorter distance and lesser ETX. The source node will construct a list of forwarder nodes based on priority. The forwarder nodes will use the list to transmit packets via end-to-end transmission. The list is maintained by each of the forwarder nodes that participated in the packet transmission. According to the authors, this method can increase the throughput of large unicast transmissions in multi-hop wireless sensor networks. However, this method produced high overhead, especially during the coordination of all the relay nodes in the network.

Mao et al. (2011) proposed a forwarder method named Energy-Efficient Opportunistic Routing (EEOR). The authors introduced a method to select a forwarder node by calculating the cost and energy using Eq. (1).

(1) \begin{eqnarray*}{C}_{u}({FWD}^{\ast })={C}_{u}^{h}({FWD}^{\ast })+{C}_{u}^{f}({FWD}^{\ast })+{C}_{u}^{C}({FWD}^{\ast })\end{eqnarray*}

*C*_*u*_(FWD^∗^) is denoted as the expected cost of a source node to broadcast a packet to the destination node. ${C}_{u}^{h}$(FWD^∗^) is the cost for determining the relay node. ${C}_{u}^{f}$(FWD^∗^) is the cost for determining the forwarder node. ${C}_{u}^{C}$(FWD^∗^) is the communication cost for the forwarder node to transmit packets. The cost of ${C}_{u}^{C}$(FWD^∗^) is incurred when the network is at the “static” mode. In “active” mode, the cost of forwarding the packets is calculated based on the traffic flows. After calculating the *C*_*u*_(FWD^∗^), this method will select the related relay nodes that have the minimum costing to forward the packets from the source node to the destination node. According to the authors, this method can minimize energy consumption and improve network lifetime. However, this method uses unicast and single-path to transmit packets. Moreover, it required the nodes that take part in the transmission to be in an “active” mode always. As a result, this method might produce high first dead node for the networks.

Lee & Haas (2011) proposed a forwarder method named Short-haul Multi-hop (Short-Haul). This method uses the shortest route between the source node and the destination node to select forwarder nodes. In this method, a source node will firstly broadcast a message to all the relay nodes. The relay node that has the closest distance to the destination node will be selected as the forwarder node to transmit packets. The selected forwarder node will send an acknowledgement to the source node once the packets have successfully received. After that, the forwarded node will broadcast a message to all the relay nodes, and then it will forward the packets to the relay node that has the closest distance to the destination node. The process of searching the closest relay nodes will be repeated until the packets have been delivered to the destination node. This method uses multi-path routing to forward the packets to the destination node. During the transmission, the sender node will retransmit the packets if it does not receive an acknowledgement from the forwarder node. However, the forwarder nodes and the destination node will discard any duplicate packets sent by the sender node. Once the destination node has received the packets, it will send an acknowledgement to all the forwarder nodes in the path. According to the author, this method is simple and can be easily integrated with other opportunistic routing algorithms. Moreover, this method can reduce the packet’s duplication problem and increase the throughput of the transmission. However, this method might consume more energy. It might produce low network lifetime because the sender nodes are required to broadcast a message to all the relay nodes for determining which relay node has the closest distance to the destination node.

In 2015, Luo et al. (2015) proposed a forwarder method named Energy Savings Via Opportunistic Routing (ENS_OR). This method uses a single-path to transmit packets. To select the forwarder node, it uses distance and energy level as in Eq. (2).

(2) \begin{eqnarray*}P \left( h+i \right) = \left\{ \begin{array}{@{}l@{}} \displaystyle \left( {d}_{h+i}-{d}_{h} \right) \left[ \frac{1}{ \left\vert {d}_{h+i}-{d}_{op} \right\vert } +{E}_{h+i}-\zeta \right] \\ \displaystyle \left( h+i \right) \in F \left( h \right) ,-R\leq i\leq R \end{array} \right. \end{eqnarray*}

P(h) is denoted as the current forwarder node. i is denoted as an integer starting from 1. (d _h+i_ − d _h_) is denoted as the distance between P(h) and P(h + i). E _h+i_ signifies the remaining energy of P (h + i). ζ signifies the value of the threshold energy. F(h) is denoted as the selected forwarder list of P(h). R signifies the maximum transmission range.

The source node will construct a list based on the acceptable distance and energy level. This list will become the priority list when selecting a relay node to forward packets. Once the path has determined, the packets will be sent via end-to-end transmission. According to the authors, this method can reduce energy consumption and increase the network lifetime. However, this method might decrease the network performance when the single-path is congested, or the relay node has insufficient energy to forward packets through the end-to-end transmission.

Raman & Sharma (2017) proposed a forwarder method named Energy Optimization Opportunistic Routing (ENO_OR). This method uses energy level and distance to select a forwarder node. Initially, the default threshold energy level is pre-configured. Any relay node that reaches the default threshold energy level will have a chance to be selected the forwarder node. However, priority will be given to the relay node that has the highest energy level and optimal distance. The optimal distance is determined using Eq. (3).

(3) \begin{eqnarray*}{D}_{op}= \frac{M-{x}_{h}}{{n}_{op}} ={ \left\{ \frac{2{E}_{a}}{ \left[ \left( \varphi -1 \right) {E}_{\mathrm{\beta }} \right] } \right\} }^{ \frac{1}{\varphi } }\end{eqnarray*}

*D*_*op*_ *is denoted as the optimal transmission distance. M is denoted as the position of the forwarder node. x*_*h*_ *is denoted as the position of the relay node. n is the index of the relay node. E*_*β*_ *is denoted as the energy required for the packet transmission. φ is denoted as the transmission loss due to link failure.*

According to the author, this method can increase the network lifetime by using the pre-configured energy threshold level and optimal distance. However, if the available relay nodes do not meet the minimum pre-configured threshold energy level, this method will use direct packets transmission to transmit packets to the destination node which might consume more energy.

Hasnain, Malik & Aydin (2018) proposed a forwarder method named Adaptive Opportunistic Routing (AOR). In this method, the forwarder node is selected based on minimum energy consumption and the link quality. In order to deliver the packets from the source node to the destination node, this method uses optimal route selection. To select the optimal route, it uses minimum energy consumption and maximum link quality. Energy consumption is calculated based on the size of the packet delivered and the distance covered from the source node to the forwarder node. To determine the maximum link quality for forwarding the packets, this method uses the probability. Equation (4) shows the formula used to calculate energy consumption.

(4) \begin{eqnarray*}{E}_{t} \left( P,d \right) = \left\{ \frac{p \left( {E}_{e}+{\gamma }_{fs}~x~{d}^{2} \right) if~d\leq {R}_{c}}{p \left( {E}_{e}+{\gamma }_{mp}~x~{d}^{4} \right) if~d\geq {R}_{c}} \right. \end{eqnarray*}

*E*_*t*_ *is denoted as the transmission range. P is denoted as the packet size. E*_*e*_ *is denoted as the overall energy consumption for the packet transmission. γ*_*fs*_ *is denoted as the forwarder node location. d*^2^ *is donated as an ideal range where packets can be successfully transmitted. γ*_*mp*_ *is denoted as the distance between the source node and the relay node. R*_*c*_ *is denoted as the maximum range to select the forwarder node.*

Equations (5) and (6) are used to calculate the link quality. These two equations are used to calculate the probability of the forwarding packets via a particular route and the progress of the forwarded packets at the route respectively.

(5) \begin{eqnarray*}{PADV(r)=ADV(r)}_{\mathrm{x}}~prob~({r}_{S}^{D},P)\end{eqnarray*}

(6) \begin{eqnarray*}ADR(r)=D(S,D)-D(S,R)\end{eqnarray*}

*PADV is donated as the probability of the packet delivery through route r, which is established based on the broadcast messages among the relay nodes.* $prob \left( {r}_{S}^{D},P \right) $ *is denoted as the probability of the successful packets delivered from the source node to the destination node. ADV(r) is donated as the progress of the forwarded packets thought route r. D(S,D) is donated as the total distance from the source node to the destination node. D(S,R) is donated as the distance of the possible forwarder node from the source node.*

According to the authors, this method can minimize energy consumption when forwarding the packets using the optimal route selection. However, this method only uses single-path and end-to-end transmission. As a result, it might increase the latency and end-to-end delay when the relay node has insufficient energy or the link quality is poor.

Khan et al. (2018) proposed a forwarder method named Cooperative Energy Efficient Optimal Relay Selection (Co-EEORS). This method produces reliable packet delivery. The forwarder node is selected based on the lowest depth and the value of the lowermost location. The value of the location interfaces and measures the distance between the source node and the destination node. Relay nodes that are located closest to the destination node will have a smaller value of the location. A relay node is selected as a forwarder node to forward the packets if it is closest to the destination node. The destination node sends an acknowledgement to the source node after receiving the packets successfully.

According to the author, the proposed method achieved a higher receiving packets ratio as compared to other forwarder methods. However, there is a limitation with regards to the performance of Co-EEORS, and this seems an exceptional condition. It occurs when there is a larger distance between the relay nodes, and when the source nodes can not find the forwarder nodes, thus cooperation fails due to link failure. As a result, this method could increase overhead and latency.

Li et al. (2019) proposed a forwarder method named Multi-hop Wireless Networks (MWN). In this method, the forwarder node is selected using energy-efficient metric. The energy-efficient metric is comprised of several parameters such as one-hop distance (*R*_1__,*t*_)*,* transmission range (*R*_2__,*t*_), and the distance between the relay node and the destination node (*d*_*t*_). The average forwarding distance and the total energy consumption are calculated for each hop using Eq. (7) and the energy consumption for each hop is calculated using Eq. (8).

(7) \begin{eqnarray*}D(n)={\mathit{min}}_{1\leq i\leq n} \left\{ {d}_{i} \right\} \end{eqnarray*}

*d*_*i*_ *is denoted as the distance between the relay node i and the destination node. If the relay node i successfully decodes the packet, d*_*i*_ *will be given a value equals to the Euclidean distance from i to the destination node. Otherwise, d*_*i*_ *will be given a value equal to d*_*t*_*.*

(8) \begin{eqnarray*}{E}_{all} \left( {R}_{1,t},{R}_{2,t} \right) = \left[ {E}_{1}+{E}_{ct}+{S}_{tp}{E}_{cr} \right] \ast L,\end{eqnarray*}

*R is denoted as the radius of the relay node. E*_1_ *is denoted as the packet transmitting energy per bit. S*_*tp*_ *is denoted as the size of the forwarding area. E*_*ct*_ *and E*_*cr*_ *are denoted as the transmitter and the receiver circuit energy consumption per bit for each relay node respectively.*

According to the authors, the use of the energy-efficient metrics can optimize the average forwarding distance with minimum energy consumption. However, the forwarder node in this method is selected dynamically. As a result, the selected forwarder nodes might have insufficient energy to forward the packets. When this situation happens, the packet receiving ratio will be decreased.

Chithaluru, Tiwari & Kumar (2019) proposed a forwarder method named adoptive ranking based energy-efficient opportunistic routing (AREOR). This method uses single path and end to end transmission to transmit the packet from source node to destination node. It selects the best relay node to take an interest as a cluster head by utilizing versatile participatory criteria. Forwarder node is selected based on an adoptive ranking system. Relay nodes ranking is determined by computing the remaining energy and location closest to the destination node.

According to the author, this method reduces energy consumption by using the adoptive ranking and optimal energy node selection. However, in this method, the forwarder node is selected based on the cluster and adoptive ranking system. Network performance will be decreased if available nodes have insufficient energy to forward the packets.

Zhang et al. (2019) proposed a forwarder method named shortest-latency opportunistic routing in asynchronous WSNs. This method theoretically examines the techniques on how to select a forwarder node in Asynchronous Wireless Sensor Networks (WSNs). The proposed approach develops the probability for the relay node to be selected as a forwarder node. This method uses single-path and bop-by-hop transmission to transmit packets from source node to destination node.

According to the author, end-to-end latency for opportunistic routing in asynchronous WSNs is theoretically achieved in this approach. However, this method might decrease network performance and to determine the real implication of this approach in terms of energy efficiency, there is a need to implement the proposed approach practically.

Wang (2020) proposed a three-layer framework is used multiple mobile sinks with fog structure. The proposed framework aims to break the bottleneck of data collection from WSNs to the cloud. The framework was compared with various existing traditional solutions. The experimental result reveals that the framework can help in the improvement of throughput and the reduction of transmission delay. Liang (2020) proposed a reliable trust computing mechanism (RTCM). The framework helps in enhancing the reliability and efficiency of data transfer to the cloud. The result shows some promise.

Thakkar and Kotecha proposed a routing algorithm that utilizes the energy-delay index for a trade-off to optimize both objectives-energy and delay (Thakkar, 2014a; Thakkar, 2014b). The result shows that the proposed algorithm performs well. Thakkar and Kotecha further proposed a cluster formation technique with a decentralized cluster head election method (Thakkar, 2015). The authors used Bollinger Bands to elect a cluster head. The result shows significant improvement. In another study by Thakkar, the author proposed an advanced LEACH protocol named DEAL (Thakkar, 2017). The protocol takes energy and distance of a node into consideration during cluster head election process. The result shows that the proposed protocol enhances the stability period in comparison to the existing state-of-the-art. Furthermore, Thakkar also published two more studies in the research theme (Thakkar, 2014a; Thakkar, 2014b; Thakkar, 2016).

Table 1 shows the synthesis of the selected related work. From the review, we noticed that most of the existing forwarder methods produce high first dead node, high latency, high energy consumption, and less network lifetime. several reasons cause these weaknesses to happen. For example, some of the forwarder methods do not consider energy level, and they always need to broadcast messages to all the relay nodes when determining the forwarder node. As a result, these types of forwarder methods might cause the relay node to die quickly or drop the received packet due to insufficient energy. Moreover, some of the forwarder methods use unicast, single-path, and end-to-end transmission to transmit packets. These types of transmissions might increase the latency and end-to-end delay when the relay node has insufficient energy, or the link quality is poor, or the path is congested.

**Table 1:** Comparison of selected related works.

**Forwarder method**	**Year**	**Routing mechanism**	**Forwarding list selection**	**Advantages**	**Disadvantages**
GeRaF (Zorzi & Rao, 2003)	2003	Multi-path	Hop-by-Hop	■ Reduce latency.	■ Decrease network lifetime.
ExOR (Biswas & Morris, 2005)	2005	Single-path	End-to-End	■ Increase throughput.	■ Increase overhead.
EEOR (Mao et al., 2011)	2011	Single-path	End-to-End	■ Minimize energy consumption. ■ Increase network lifetime.	■ Produce high first dead node.
Short-Haul (Lee & Haas, 2011)	2011	Multi-Path	Hop-by-Hop	■ Increase throughput ■ Reduce the ratio of the duplication packets.	■ Produce high energy consumption. ■ Decrease network lifetime.
ENS_OR (Luo et al., 2015)	2015	Single-path	End-to-End	■ Minimize energy consumption. ■ Increase network lifetime.	■ Decrease network performance.
ENO_OR (Raman & Sharma, 2017)	2017	Single-path	Hop-by-Hop	■ Increase network lifetime.	■ Consume more energy.
AOR (Hasnain, Malik & Aydin, 2018)	2018	Single-path	End-to-End	■ Minimize energy consumption.	■ Increase latency. ■ End-to-end delay.
Co-EEORS (Khan et al., 2018)	2018	Single-path	End-to-End	■ Increase receiving packets ratio.	■ Increase overhead. ■ Increase latency.
MWN (Li et al., 2019)	2019	Single-path	Hop-by-Hop	■ Minimize energy consumption.	■ Decrease receiving packets ratio.
AREOR (Chithaluru, Tiwari & Kumar, 2019)	2019	Single-path	End-to-End	■ Reduce energy consumption.	■ Decrease network performance.
Shortest-Latency (Zhang et al., 2019)	2019	Single-path	Hop-by-Hop	■ Reduce latency.	■ Decrease network performance.
Wang et al. (Wang, 2020)	2020	Single-path	Hop-by-Hop	■ Minimize energy consumption.	■ Increase latency.
Liang et al. (Liang, 2020)	2020	Multi-Path	Hop-by-Hop	■ Increase throughput.	■ Decrease network lifetime.
Thakkar and Kotecha (Thakkar, 2014a; Thakkar, 2014b)	2014	Multi-Path	Hop-by-Hop	■ Increase network lifetime. ■ Reduce the ratio of the duplication packets.	■ Decrease network lifetime.
Thakkar and Kotecha (Thakkar, 2015)	2015	Multi-Path	Hop-by-Hop	■ Minimize energy consumption. ■ Reduce latency.	■ Decrease network performance.
Thakkar (Thakkar, 2017)	2017	Single-path	End-to-End	■ Minimize energy consumption.	■ Increase latency.
Thakkar and Kotecha (Thakkar, 2014a; Thakkar, 2014b)	2014	Multi-Path	Hop-by-Hop	■ Minimize energy consumption. ■ Reduce latency.	■ Decrease network performance.
Thakkar (Thakkar, 2016)	2016	Multi-Path	Hop-by-Hop	■ Minimize energy consumption. ■ Reduce latency.	■ Decrease network performance.

From this review results, it is shown that there is still room for improvement in this domain, especially in the aspects of latency, first dead node, network lifetime, and receiving packets ratio. Thus, this research was carried out to propose a relay node selection method to improve the drawbacks above.

## Materials & Methods

In this section, the simulation settings used by Luo et al. (2015) to evaluate the proposed method are presented. The simulation is conducted in OMNET++ simulator and MiXiM framework. The proposed method and the related works are simulated using the simulation settings in Table 2. OMNET++ and MiXiM are chosen because they have the required libraries such as stdio.h, string.h, omnetpp.h, “bs.h”, “node.h”, “cl_msg_m.h”, “gesteb.h”, and c0utVector class which are required when implementing the proposed method and the related works (Bouachir et al., 2016; Zhao, Mosler & Braun, 2012). The simulation is carried out in an area of 500 m2 network size with 100 nodes that are uniformly deployed. The network consists of one source node, one destination node, and 98 relay nodes. The maximum range between relays nodes is 30 m, while the minimum range is 15 m. The packet size 1,024 bit is used for transmission. The initial threshold energy level is set as 50%. The sending rate is one packet per second. The simulation time is set 900 s and adopted from Luo et al. (2015). The simulation is executed 100 times for each result, as suggested by Ritter et al. (2011). The simulation results are collected individual and manually, and “R” program is used to compare the results

**Table 2:** Simulation settings.

**Parameter**	**Values**
Network size	500 m ×500 m
Node deployment	Uniform
Number of nodes	100
Source node	1
Destination node	1
Relay nodes	98
Maximum range	30 m
Minimum range	15 m
Packet size	1,024 bit
Threshold energy level	50%
Sending rate	1 packet/s
Simulation time	900 s

### Proposed methods

To provide a clear overview, a high-level description of the proposed method is described in this section. To ease the explanation, we pre-configured the threshold energy and the energy level for each relay node before demonstrating how a given packet is sent from the source node to the destination node. Initially, the source node will select a relay node to forward a packet based on the distance and the energy level. In the previous studies, some methods used either distance or energy level to perform routing. Moreover, several researchers (Hawbani et al., 2019; Kannan & Raja, 2015; Nadar et al., 2017) reported that distance and energy levels are the most commonly used metrics to select the best relay node. Therefore, we used and improvised these two metrics in our proposed method to select a relay node. In our proposed method, the selected relay node is called the forwarder node. The source node will forward the packet to the forwarder node. After transmitting the packet, the energy level of the source node will be reduced based on the distance covered and the size of the packet delivered during the transmission. The current forwarder node will use the same mechanism to select another relay node to become the next forwarder node.

Similarly, the energy level of the current forwarder node will be reduced after the packet is transmitted to the next forwarder node. This process will be repeated until the packet reaches the destination node. Hence, the proposed method has a distributed architecture.

### Proposed method illustration

**Figure 1:**
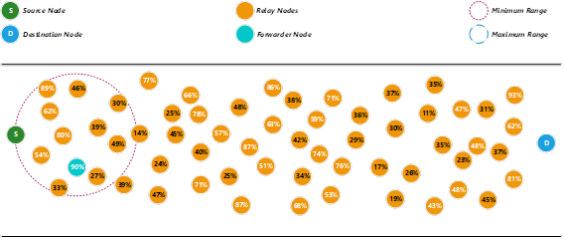
Determine a forwarder node using the minimum range.

Assuming that a source node (S) is going to transmit a packet to a destination node (D), and the pre-configured threshold energy level is set as 50%. In our proposed method, S will first use the minimum range to search for an available relay node to become the forwarder node (see Fig. 1). Equation (9) is adopted from Alia & Al-Ajouri (2016) to calculate the minimum range.

(9) \begin{eqnarray*}DminR \frac{min \left( i,l \right) d \left( si,sl \right) }{\sqrt{{W}_{m}^{2}+{H}_{m}^{2}}} \end{eqnarray*}

$min \left( i,l \right) d \left( si,sl \right) $ *is the minimum distance between relay nodes.* $\sqrt{{W}_{m}^{2}+{H}_{m}^{2}}$ *is the maximum length between any two relay nodes which can be represented by the diagonal length of the monitored field.*

Since there is more than one relay node with a threshold energy level more than or equal to 50%, therefore, the proposed method will give higher priority to the relay node that has the highest energy level. If there is a tie, the nearest distance will become the second priority for the selection process. If there is no relay node fulfils the minimum threshold energy level (50%), the proposed method will use the maximum range to search for any suitable relay nodes to become the forwarder node. In this example, the relay node that has a 90% energy level is selected as the forwarder node.

**Figure 2:**
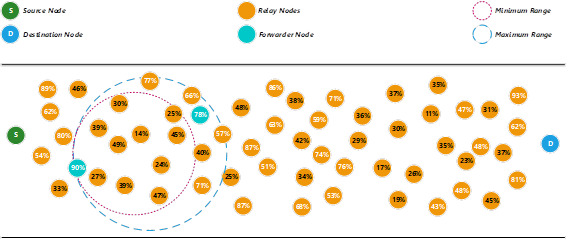
Determine a forwarder node using the maximum range.

To find the next forwarder node, the proposed method will use the minimum range to search for an available relay node that fulfils the minimum energy level threshold (50%). Since there is no relay node fulfils the minimum energy level threshold, the proposed method then uses the maximum range to search for any suitable relay nodes to become the next forwarder node (see Fig. 2). Equation (10) is adopted from Luo et al. (2015) to calculate the maximum range.

(10) \begin{eqnarray*}{d}_{op}=M-{x}_{h}={ \left\{ \left( 2{E}_{elec} \right) / \left[ \left( \tau -1 \right) {\varepsilon }_{amp} \right] \right\} }^{1/\tau }\end{eqnarray*}

*d*_*op*_ *is the optimal transmission distance. M is the index of a relay node. x*_*h*_ *is the position of the relay nodes. E*_*elec*_ *is the energy consumption of the relay node during transmission.* ε_*amp*_ *is the energy dissipated in the transmit amplifier. τis the channel route-loss exponent of the antenna. d is the distance between the current forwarder node and the next forwarder node.*

In this example, the relay node that has 78% energy level is selected as the next forwarder node. The energy level of the previous forwarder node will be reduced after the packet is transmitted to the next forwarder node.

**Figure 3:**
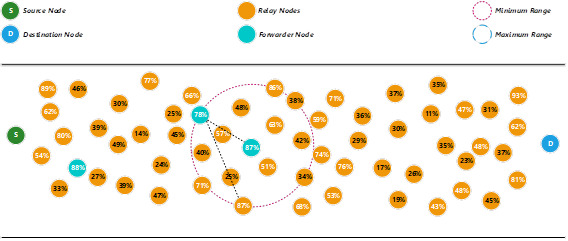
Determine a forwarder node using the nearest distance.

To find the next forwarder node, the same mechanism is used. The proposed method will first use the minimum range to search for an available relay node that fulfils the minimum threshold energy level. Since there are two relay nodes with the same energy level (87%), the nearest distance will become the second priority for the selection process (see Fig. 3). In this example, the relay node that is closest to the current forwarder node is selected as the next forwarder node. The energy level of the previous forwarder node will be reduced after the packet is transmitted to the next forwarder node.

**Figure 4:**
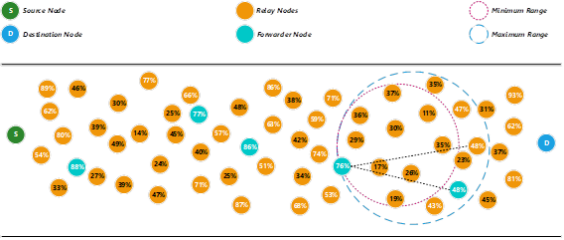
Determine a forwarder node by decreasing the threshold energy level.

The find next forwarder node, the proposed method will use the minimum range to search for an available relay node that fulfils the minimum threshold energy level (see Fig. 4). Since there is no relay node fulfils the minimum threshold energy level, the proposed method then uses the maximum range to search for any suitable relay nodes to become the next forwarder node. Since there is no relay node fulfils the maximum threshold energy level; also, the proposed method will reduce the threshold energy level using Eq. (11).

(11) \begin{eqnarray*}T{h}_{energy}= \left\{ T{h}_{energy}x \frac{Ere}{Ein} \right. i{f}_{elsewire}^{n\in G}\end{eqnarray*}

*Th*_*energy*_ *is the threshold for the energy level, Ere is the residual energy of the relay node. Ein is the initial energy of the relay node. G is the set of all the relay node.*

Assuming that the new threshold energy level after the calculation is 40%. A broadcast message will be sent to notify each of the relay nodes about the new threshold level. The proposed method then continue using the minimum range to search for an available relay node that fulfils the new threshold energy level. In this example, the relay node that has the highest energy level is selected as the next forwarder node. The energy level of the previous forwarder node will be reduced after the packet is transmitted to the next forwarder node.

**Figure 5:**
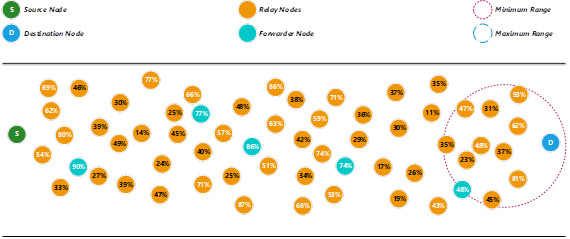
Transmit a packet to the destination node.

The current forwarder node continues to search for the next available relay node within the minimum range to forward the packet. Since the destination node is within the minimum range, the packet will deliver to the destination node (see Fig. 5).

**Figure 6:**
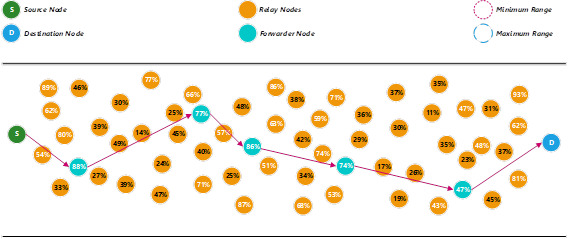
Paths of the packet transmitted using the proposed method.

**Figure 7:**
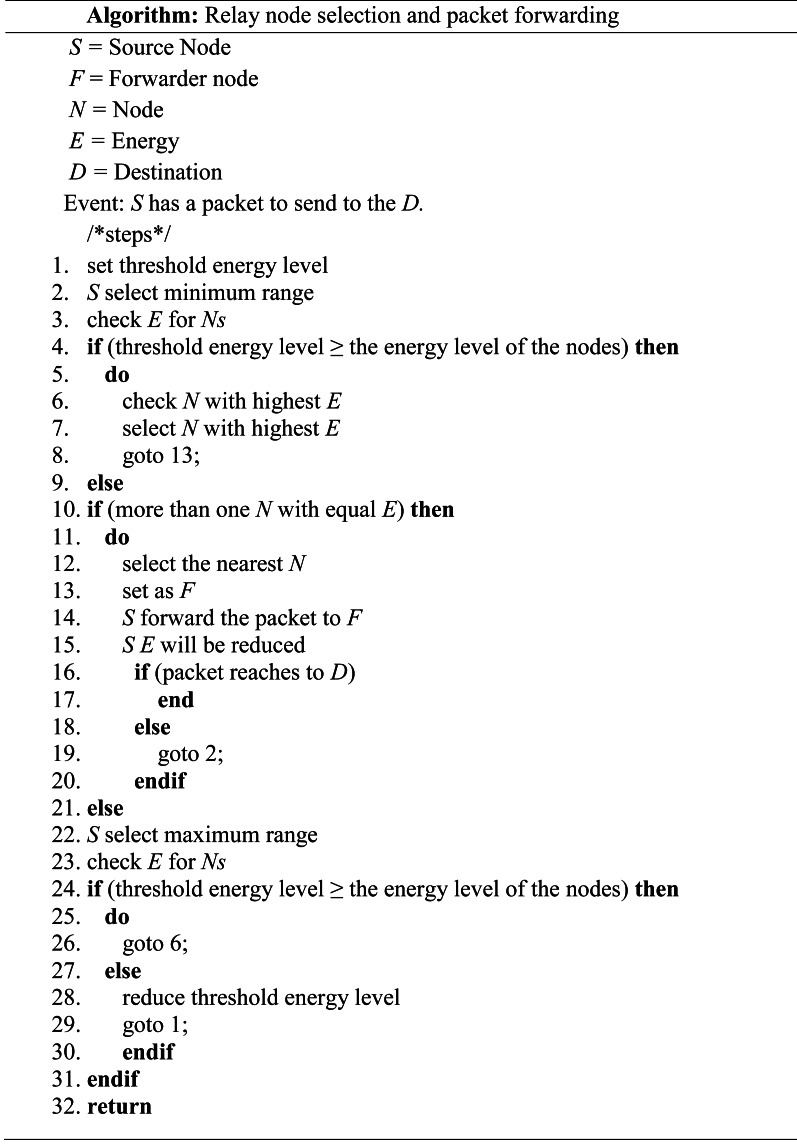
The pseudo code of the proposed method.

**Figure 8:**
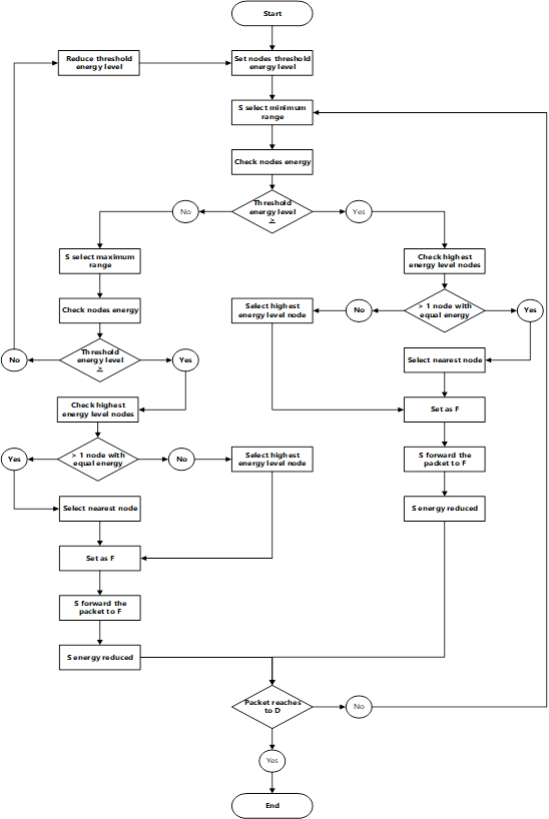
Flowchart of the proposed method.

In this example, a packet only required six hops to transmit from a source node to a destination node using the proposed method (see Fig. 6). The pseudo-code and the flowchart of the proposed method are shown in Figs. 7 and 8, respectively.

## Results

The proposed method and other related works are evaluated based on the following routing metrics:

**Latency (L):** L is used to determine the average time of the packets that are successfully delivered to the destination node.

**First dead node (FDN):** FDN is defined to measure the network connectivity and to check the appearance of the first dead node in the network.

**Network lifetime (NL):** NL is used to determine the energy consumption and network partition. FDN and NL are essential metrics to increase the network lifetime.

**Receiving packets ratio (RPR):** RPR is used to determine the total number of packets that are successfully received by the destination node.

These aspects are used because this is the main focus of our research work. Moreover, the other related works also used the same metrics for evaluation (Hasnain, Malik & Aydin, 2018; Luo et al., 2015; Raman & Sharma, 2017). Therefore, we believe the evaluation and comparison can be carried out fairly. The details of each result analysis are discussed in the following subsections.

### Result analysis for latency (L)

**Figure 9:**
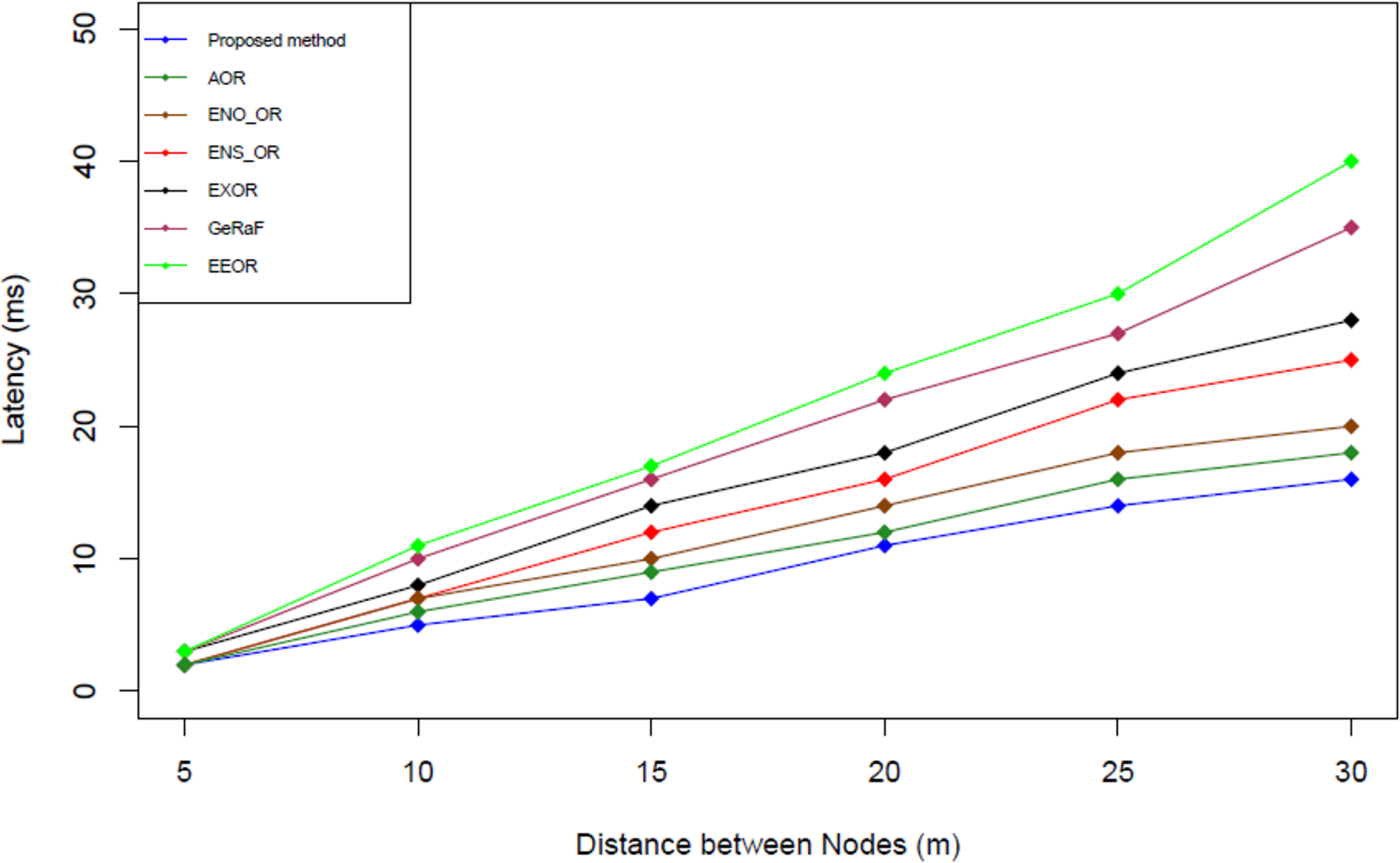
Latency comparison.

Figure 9 illustrates the packet delivery latency comparison among the proposed method and the other related works. Packet delivery latency is calculated based on the formula used by Liang, Luo & Xu (2013) (see Eq. (12)). This equation is used because it is the standard formula used to calculate the latency (L) in the opportunistic network (Liang, Luo & Xu, 2013).

(12) \begin{eqnarray*}L=\sum _{i=0}^{n} \left( {T}_{contention} \left( k \right) +{T}_{data} \right) + \left( N-1 \right) \end{eqnarray*}

${\mathop{\sum }\nolimits }_{i=0}^{n} \left( x \right) $ *is the summation for all relay nodes, x is the parameters, T*_*contention*_*(k) is the contention time, T*_*data*_ *is the packet transmission time, N is the number nodes.*

The simulation results shown that our proposed method has the lowest packet delivery latency followed by Adaptive Opportunistic Routing (AOR), ENergy Optimization Opportunistic Routing (ENO_OR), ENergy Savings via Opportunistic Routing (ENS_OR), Opportunistic multi-hop routing (ExOR), Geographic Random Forwarding (GeRaF) and Energy-Efficient Opportunistic Routing (EEOR). On average, our proposed method produces approximately 23.38%, 27.57%, 32.56%, 43.48%, 64.15%, and 75.86% lesser packet delivery latency compared to AOR, ENO_OR, ENS_OR, ExOR, GeRaF and EEOR respectively. The reason that our proposed method could perform better compared to other methods might due to the minimum or maximum range selection mechanism used. In the best case scenario, if all the forwarder nodes fulfilled the required threshold energy and used the minimum range to forward the packets, the packets could be delivered without further delay.

### Result analysis for first dead node (FDN)

**Figure 10:**
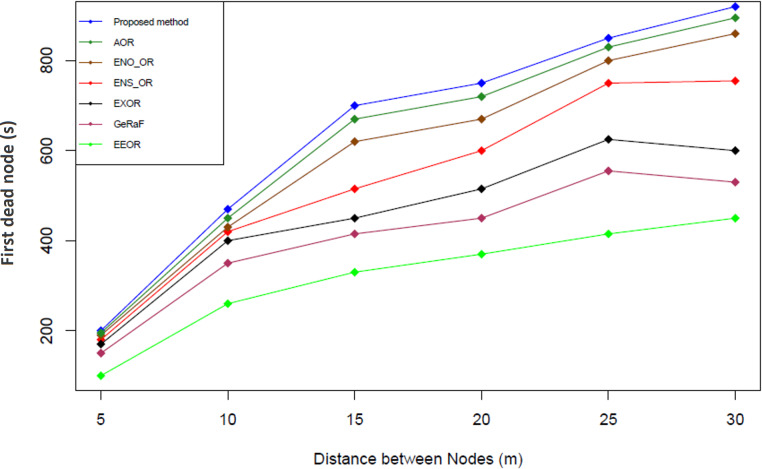
First dead node comparison.

Figure 10 illustrates the first dead node comparison among our proposed method and the other related works. First dead node is calculated based on the formula used by Ren et al. (2016) (see Eq. (13)). This equation is used because it is the standard formula used to calculate the first dead node (FDN) in the opportunistic network (Ren et al., 2016).

(13) \begin{eqnarray*}FDN= \left[ \frac{{E}_{0}}{ma{x}_{Ex}^{ \left( 0 \right) }} \right] \end{eqnarray*}

*E*_0_ *is the initial energy of the relay node.* $ma{x}_{Ex}^{ \left( 0 \right) }$ *is the maximum energy consumption of the relay node.*

The simulation results shown that our proposed method has the highest simulation time for the first dead node followed by AOR, ENO_OR, ENS_OR, ExOR, GeRaF and EEOR. On average, our proposed method produces approximately 10.68%, 15.54%, 17.14%, 42.11%, 50.30%, and 68.61% longer simulation time for the first dead node compared to AOR, ENO_OR, ENS_OR, EXOR, GeRaF and EEOR respectively. The reason that our proposed method could perform better compared to other methods might due to the optimum energy level selection mechanism used. Averagely, in our proposed method, if a relay node was selected to forward a packet, it normally would not be selected again in the subsequence round to forward a packet unless it has the highest energy level among the other relay nodes and still fulfill the required threshold energy level. Therefore, our proposed method could prolong the time of the first dead node at the same time we could still select the best relay node to forward the packet.

### Result analysis for network lifetime (NL)

**Figure 11:**
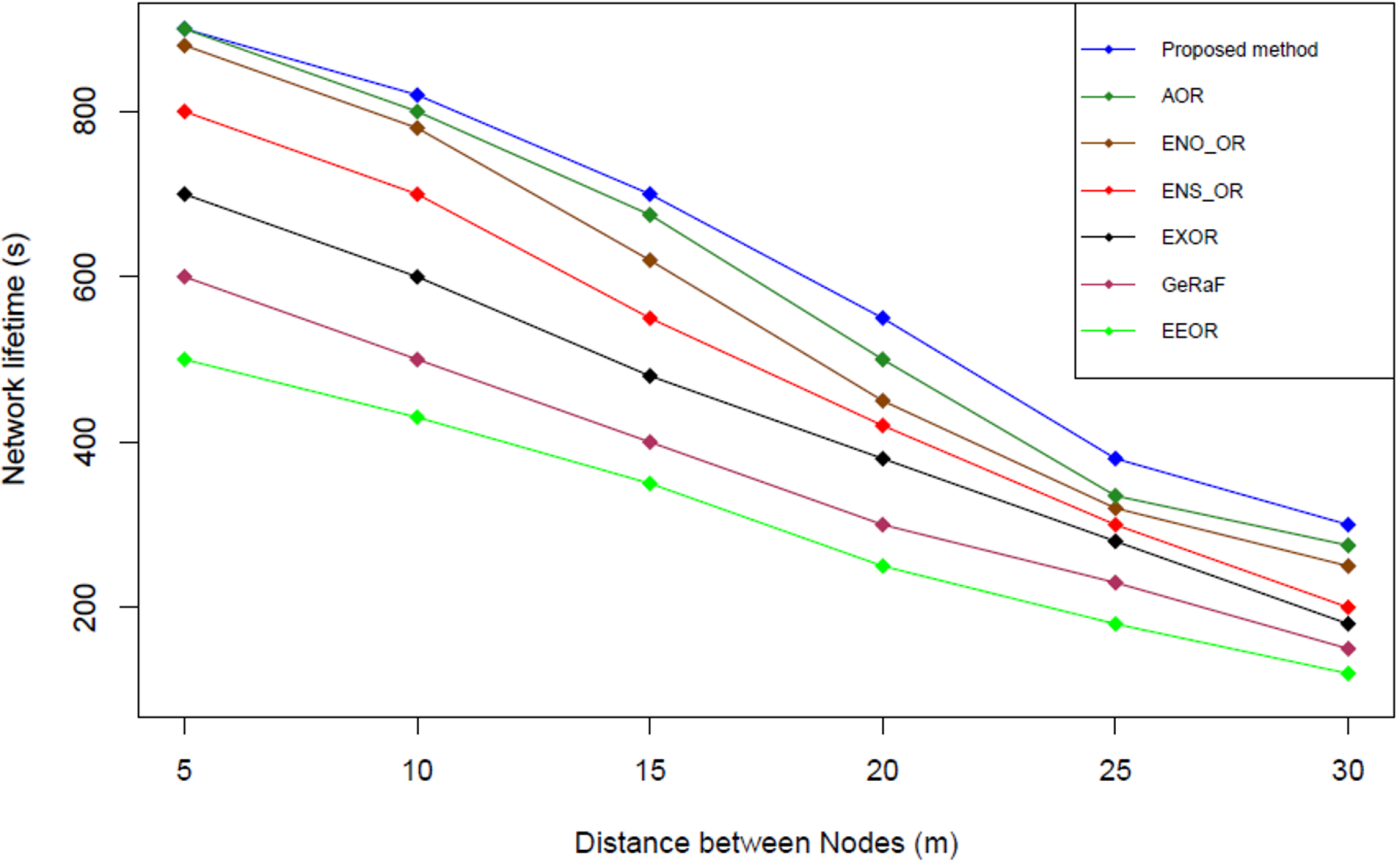
Network lifetime comparison.

Figure 11 illustrates the network lifetime comparison among our proposed method and the other related works. Network lifetime is calculated based on the formula used by Ren et al. (2016) (see Eq. (14)). This equation is used because it is the standard formula used to calculate the network lifetime (NL) in the opportunistic network (Ren et al., 2016).

(14) \begin{eqnarray*}NL=n{E}_{0}-\sum _{c=0}^{i}\sum _{j=0}^{n} \left( {E}_{j}^{ \left( i \right) }\ast {l}^{ \left( i \right) } \right) \end{eqnarray*}

*nE*_0_ *is the initial energy of the network,* ${\mathop{\sum }\nolimits }_{c=0}^{i}{\mathop{\sum }\nolimits }_{j=0}^{n} \left( {E}_{j}^{ \left( i \right) }\ast {l}^{ \left( i \right) } \right) $ *is the remaining energy of the network.* ${E}_{j}^{ \left( i \right) }$*is the average energy consumption of the relay node.* ${l}^{ \left( i \right) }$ *is the duration of the relay node.*

The simulation results shown that our proposed method has the highest network lifetime followed by AOR, ENO_OR, ENS_OR, ExOR, GeRaF and EEOR. On average, our proposed method produces approximately 7.10%, 8.35%, 10.58%, 28.57%, 50%, and 66.67% higher network lifetime compared to AOR, ENO_OR, ENS_OR, EXOR, GeRaF and EEOR respectively. The reason that our proposed method could perform better compared to other methods might due to the selection mechanism used in our proposed method that based on nearest distance followed by the highest energy level. Moreover, our proposed method could reduce the threshold energy level if a suitable relay node could not be found after using the minimum/maximum range as well as the existing threshold energy level. Therefore, our proposed method could prolong the network lifetime.

### Result analysis for receiving packets ratio (RPR)

**Figure 12:**
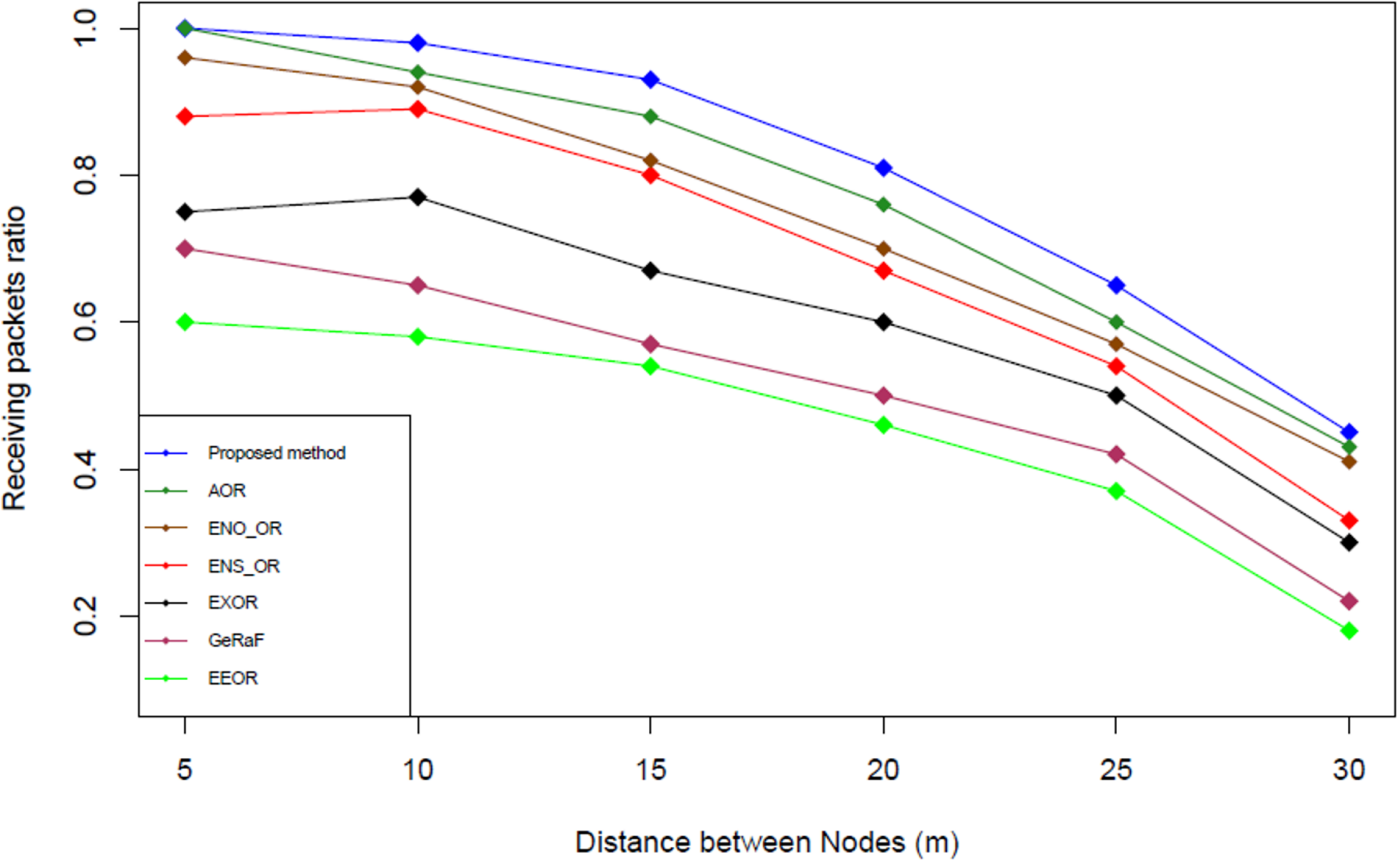
Receiving packets ratio comparison.

Figure 12 illustrates the receiving packets ratio comparison among the proposed method and the other related works. Receiving packets ratio is calculated based on the formula used by Ren et al. (2016) (see Eq. (15)). This equation is used because it is the standard formula used to calculate the receiving packets ratio (RPR) in the opportunistic network (Ren et al., 2016).

(15) \begin{eqnarray*}RPR=1- \frac{\sum RDP}{\sum SDP} \end{eqnarray*}

∑*RDP is the total number of packet received by the destiation node.* ∑*SDPis the total number of packet sent to the destinaton node.*

The simulation results shown that our proposed method has the highest receiving packet ratio followed by AOR, ENO_OR, ENS_OR, ExOR, GeRaF and EEOR. On average, our proposed method produces 21.55%, 27.77%, 30.67%, 40%, 68.66%, and 85.71% higher receiving packets ratio compared to AOR, ENO_OR, ENS_OR, EXOR, GeRaF and EEOR respectively. The reason that our proposed method could produce higher receiving packets ratio compared to other methods might due to the selection mechanism used in our proposed method that based on nearest distance followed by the highest energy level. Additionally, our proposed method could reduce the threshold energy level if no suitable relay node could be found after using the minimum/maximum range as well as the existing threshold energy level.

As a whole, the simulation results shown that our proposed method was able to perform better than the related works because of the following reasons: (i) Our proposed method could use a relay node that falled within the minimum range to forward the packet if the relay node has the highest energy level. Therefore, it would reduce the latency when delivering the packet to the destination node. (ii) The optimum energy level selection mechanism used in our proposed method could reduce the chances of a forwarder node from taking part to forward a packet again unless it still has the maximum energy level compared to other relay nodes. Therefore, it will prolong the time of the first dead node. (iii) Our proposed method could reduce the threshold energy level from time to time if no suitable relay node is found. Our selection mechanism could use the new threshold energy level together with the minimum/maximum range searching mechanism to determine a suitable relay node. As a result, our proposed method could prolong the network lifetime and produce a higher receiving packet ratio.

## Conclusions and Future Work

In this paper, an improved relay node selection method was proposed. The proposed method that uses the minimum or maximum range and optimum energy level to select the best relay node to forward the packet was proposed to improve the performance of routing in the opportunistic network. In our proposed method, the threshold energy level needs to be pre-configured. After that, our proposed method will use the minimum range to determine a forwarder node. The maximum range will only be used if no relay node fulfills the minimum threshold energy level within the minimum range. To select the forwarder node, priority is given to the node that has the highest energy level. If there is a tie, the nearest distance will become the second priority for the selection process. If no relay nodes meet the minimum threshold energy level, the proposed method reduces the threshold energy level and uses the same mechanism to determine a forwarder node. A broadcast message is sent to notify all the relay nodes about the new threshold level. This process is repeated until a packet is forwarded to the destination node. Several simulations were conducted to evaluate the proposed method based on L, FDN, NL, and RPR. The results showed that our proposed method could (i) produce lower latency, (ii) prolong the time for the first dead node, (iii) improve the network lifetime, and (iv) produce a higher receiving packet ratio compared to other methods.

For future work, we intend to investigate whether it is practical to integrate our proposed method with “network coding”. Zhai et al. (2018) proposed “network coding” technique in 2018. According to the authors, “network coding” is a technique that can be used to forward more than one packet/message in each transmission. As a result, an assumption was made that by integrating the proposed method with “network coding”, the performance of routing in the opportunistic network could be improved. Therefore, in the future, more researches would be carried out in this area. Besides, we intend to investigate whether it is practical to integrate our proposed method with Cognitive Radio Networks (CRNs). Kafaie et al. (2018) proposed the CRNs technique in 2018. CRNs is a paradigm of wireless communication that allows unlicensed secondary users to adjust their transmission parameters in order to achieve efficient usage of radio spectrum resources without any harmful interference to the licensed primary user. CRNs are getting more and more popular in the opportunistic network because it provides dynamic spectrum access, and more efficient and secure data transmission (Kafaie et al., 2018). An assumption was made that by integrating our proposed method as one of the features or libraries in CRNs, it might create more revenue for researchers in this domain. However, the proposed method might not fit well in the current routing metrics in CRNs. Therefore, in the future, more researches would be carried out to enable our proposed method to be embedded as one of the feature or library in CRNs.

##  Supplemental Information

10.7717/peerj-cs.326/supp-1 Supplemental Information 1 Code of the proposed scheme.

## Abbreviations

The following abbreviations are used in this manuscript:

AOR: Adaptive Opportunistic Routing
AREOR: Adaptive Ranking based Energy-efficient Opportunistic Routing
Co-EEORS: Cooperative Energy Efficient Optimal Relay Selection
CRNs: Cognitive Radio Networks
D: Destination node
E: Energy
EEOR: Energy Efficient Opportunistic Routing
ENO_OR: Energy Optimization Opportunistic Routing
ENS_OR: Energy Savings via Opportunistic Routing
ETT: Expected Transmission Time
ETX: Expected Transmission Count
ExOR: Opportunistic Multi-hop Routing
F: Forwarder node
FDN: First dead node
GeRaF: Geographic Random Forwarding
L: Latency
MiXiM: Mixed simulator
Min-Max Range: Minimum and Maximum range
MWN: Multi-hop Wireless Networks
N: Node
NL: Network Lifetime
OMNeT++: Objective Modular Network Testbed in C++
OR: Opportunistic Routing
RPR: Receiving Packets Ratio
S: Source node
Shortest-latency: Shortest-latency opportunistic routing in asynchronous WSNs
Short-Haul: Short-haul Multi-hop
WSNs: Wireless Sensor Networks

## Additional Information and Declarations

### Data Availability

The following information was supplied regarding data availability: The code is available in the Supplemental Files.
